# Differences in sequencing technologies improve the retrieval of anammox bacterial genome from metagenomes

**DOI:** 10.1186/1471-2164-14-7

**Published:** 2013-01-16

**Authors:** Fabio Gori, Susannah G Tringe, Gianluigi Folino, Sacha AFT van Hijum, Huub JM Op den Camp, Mike SM Jetten, Elena Marchiori

**Affiliations:** 1 Radboud University Nijmegen, Institute for Computing and Information Science, Heyendaalseweg 135, 6525 AJ Nijmegen, The Netherlands; 2 DOE Joint Genome Institute, Walnut Creek, CA 94598, USA; 3 ICAR-CNR, Rende, Italy; 4 Center for Molecular and Biomolecular Informatics, Radboud University Nijmegen Medical Centre, Nijmegen, The Netherlands; 5 Department of Microbiology, Institute for Water and Wetland Research, Radboud University Nijmegen, Heyendaalseweg 135, 6525 AJ Nijmegen, The Netherlands; 6 Delft University of Technology, Department Biotechnology, 2628 BC Delft, The Netherlands

## Abstract

**Background:**

Sequencing technologies have different biases, in single-genome sequencing and metagenomic sequencing; these can significantly affect ORFs recovery and the population distribution of a metagenome. In this paper we investigate how well different technologies represent information related to a considered organism of interest in a metagenome, and whether it is beneficial to combine information obtained using different technologies. We analyze comparatively three metagenomic datasets acquired from a sample containing the anammox bacterium *Candidatus* ’Brocadia fulgida’ (*B. fulgida*). These datasets were obtained using Roche 454 FLX and Sanger sequencing with two different libraries (shotgun and fosmid).

**Results:**

In each dataset, the abundance of the reads annotated to *B. fulgida* was much lower than the abundance expected from available cell count information. This was due to the overrepresentation of GC-richer organisms, as shown by GC-content distribution of the reads. Nevertheless, by considering the union of *B. fulgida* reads over the three datasets, the number of *B. fulgida* ORFs recovered for at least 80% of their length was twice the amount recovered by the best technology. Indeed, while taxonomic distributions of reads in the three datasets were similar, the respective sets of *B. fulgida* ORFs recovered for a large part of their length were highly different, and depth of coverage patterns of 454 and Sanger were dissimilar.

**Conclusions:**

Precautions should be sought in order to prevent the overrepresentation of GC-rich microbes in the datasets. This overrepresentation and the consistency of the taxonomic distributions of reads obtained with different sequencing technologies suggests that, in general, abundance biases might be mainly due to other steps of the sequencing protocols. Results show that biases against organisms of interest could be compensated combining different sequencing technologies, due to the differences of their genome-level sequencing biases even if the species was present in not very different abundances in the metagenomes.

## Background

Metagenomics studies the genomic content of microbial communities, acquired through DNA sequencing technology [1]. The main advantage of this discipline is that it can overcome the limitations of individual genome sequencing, which requires isolation and cultivation of individual microbes. Bypassing the cultivation step, metagenomics is able to acquire microbial genomes unattainable through individual sequencing, since less than 1% of the microbes present in nature can be cultured [2].

Previous study showed that the sequencing technologies have different biases, in acquiring the DNA sequences of a microbial community and of a single organism. Indeed, biases in population distribution of a metagenome may differ according to the approach adopted to obtain sequence data [3]. Moreover, there is the possibility that key members of a community might be poorly represented in sequenced data [4]. From single DNA sample study, it was shown that different technologies can also have different biases in sequencing and hence different coverage patterns of the same sequence of an organism [5]. Even sequencing errors and artifacts depend on the technology [6].

Here we focus on the comparative analysis of metagenomic sequencing data: we investigate how well different technologies represent information related to a considered organism of interest, and whether it is beneficial to combine information obtained using different technologies. The chosen microbe, *Candidatus* ‘Brocadia fulgida’, belongs to the important bacterial group of the *anammox* bacteria. Anaerobic ammonium oxidizing (anammox) bacteria obtain energy via oxidation of ammonium to dinitrogen gas in the absence of oxygen [7]. They belong to the order *Brocadiales* within the phylum *Planctomycetes*[8-10]. Many studies in the last decade showed that anammox bacteria are present in many oxygen-limited marine and fresh-water ecosystems, and the process contributes significantly to the global loss of fixed nitrogen [11-15]. Moreover, the anammox process has been applied successfully as an environmentally friendly and cost-effective alternative to conventional wastewater-treatment plants [16,17].

The choice of an anammox bacterium as the organism of interest is motivated by the lack of genomic information for this bacterial group, due also to the difficulty of acquiring it. Among the candidate genera of anammox bacteria that have been identified [10,18,19], detailed genomic information is available only for *Candidatus* ‘Kuenenia stuttgartiensis’ [20] (henceforth referred as *Kuenenia*). Indeed, standard sequencing approaches cannot be applied to acquire the genomes of these bacteria: the cultivation of anammox bacteria is challenging due to their long generation times (2-3 weeks) and low biomass yields [18,21]; moreover, no anammox species have been isolated in pure cultures up to now [22]. Therefore metagenomics has been used for acquiring the genomic content of anammox bacteria [20].

We used the genomic information of the anammox bacterium *Candidatus* ‘Brocadia fulgida’ (henceforth referred as *B. fulgida*) as a model for comparing three single-technology approaches and the multi-technology resulting from their combination. Metagenomic data containing this bacterium were acquired through three metagenomic sequencing projects conducted on the same microbial community [23]. These metagenomes were generated by the following DNA sequencing technologies: Roche 454 FLX, Sanger sequencing with shotgun library [24,25], and Sanger sequencing with Fosmid library [26] (henceforth, we refer to these technologies as 454, Shotgun and Fosmid, respectively). We reported earlier a qualitative analysis of these metagenomes focused on anammox metabolic genes [27].

First we studied the metagenomes with respect to their taxonomic population distributions and the GC-content of the reads. Then we analyzed comparatively the sets of *B. fulgida* ORFs that were recovered by the different sequencing technologies; the recovered ORFs were compared with respect to the coverage pattern, and the percentage of covered amino acids (here called *mapping*). We also studied the ORFs with respect to their functional content and their location on the genome.

## Results and discussion

### Taxonomic annotation and GC-content analysis of annotated reads

**Figure 1:**
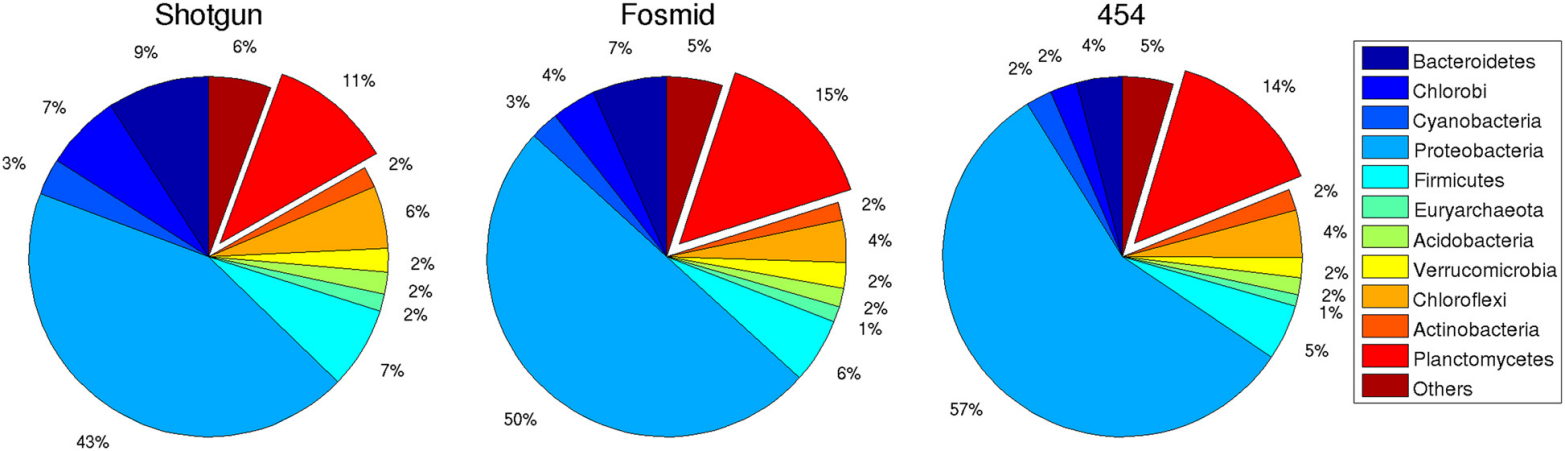
**Taxonomical annotation of reads.** Taxonomical annotation of reads at rank phylum, for different sequencing technologies.

BLASTX-based taxonomic annotation of the datasets was performed to identify the *B. fulgida* reads. Despite the metagenomes were generated with different sequencing technologies, the obtained population distributions were not very dissimilar, as shown in Figure 1. This result is consistent with that of a previous work, where the population distribution biases were shown to depend more on DNA-extraction method rather than on sequencing technology [3]; however, our metagenomic data did not allow us to verify directly this phenomenon, because the three protocols differ only from the library preparation step onward. Comparison of the population distributions with cell count estimation performed in a previous study [23] showed that *B. fulgida* was underrepresented in the sequenced data (Additional file 1: Section 1). Indeed, while *B. fulgida* constituted 70-80% of the community cells, in each dataset 11-15% of the total base pairs of the annotated reads belonged to *B. fulgida*.

**Figure 2:**
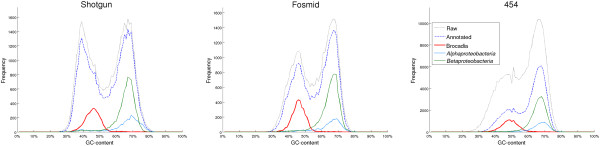
**GC-content distribution of reads.** For each technology, four GC-content distributions are shown. These correspond to the distributions obtained for: all the reads (black dotted), reads with a feasible annotation (blue dashed), reads assigned to *B. fulgida* (red) and reads assigned to classes *Alphaproteobacteria* (cyan) and *Betaproteobacteria* (green).

This gap between *B. fulgida* cell count and its abundance in the metagenomes was due to an overrepresentation of other organisms having GC-content higher than the one of *B. fulgida*. Indeed, the GC-content distribution of the reads indicated that the three datasets were biased towards GC-rich members of the community (Figure 2). In previous works it has been shown that if a bacterial genome is split into equally size non-overlapping sequences, the distribution of the GC-content of the sequences (especially for short ones) will be similar to a normal distribution centered on GC-content of the genome [28,29]. Consequently, the GC-content of reads sequenced from a single bacterium is expected to roughly follow a normal distribution and the GC-content of a metagenome could be approximately modeled by means of a mixture of normal distributions. In our case, for each technology, the distribution of the GC-content of the reads resembled the combination of two normal distributions: the one centered on GC between 38% and 50% included reads assigned to *B. fulgida*; the other one was centered between 65% and 67%. For each technology, 50% to 58% of the reads belonged to the distribution with high GC-content (GC-content above 55%) and therefore were sequenced from GC-rich bacteria. This shows that the metagenomes were biased toward GC-rich bacteria, because these microbes actually constituted less than 20-30% of the cells (70-80% of the community was made by the AT-rich *B. fulgida*). According to BLASTX, these GC-rich bacteria mostly belonged to classes *Alphaproteobacteria* and *Betaproteobacteria*.

Reads assigned to *B. fulgida* had low GC-content, consistently with their annotation. Nevertheless, a possible hypothesis is that other AT-rich reads belonging to *B. fulgida* were wrongly assigned by BLASTX to other species. However, less than 1.50% of the reads were assigned to other bacteria belonging to *B. fulgida*’s phylum - *Planctomycetes*. Moreover the population distributions obtained from different sequencing technologies were very similar; therefore, this hypothesis would require a significant difference in ORFs composition between *B. fulgida* and the other *Planctomycetes*, *Kuenenia* included. For each technology, the GC-content of the reads assigned to *B. fulgida* roughly followed a normal distribution, centered between 45% and 48%. This result is in accordance with the expected GC-content of *B. fulgida*, estimated to be close to 41%, that is, *Kuenenia*’s GC-content. However, from 42% to 50% of the reads had GC-content below 55%; since the corresponding distribution was centered between 38% and 50% of GC-content, there were other reads of this distribution with a GC-content compatible with *B. fulgida*.

In summary, these results show that GC-rich bacteria were overrepresented in the metagenomic data, for all the considered sequencing technologies. This indicates that adjustments of sequencing protocols are desirable in order to prevent overrepresentation of these microbes in the data at the expense of AT-rich *B. fulgida*. This bias toward GC-rich organisms might depend on DNA-fragmentation procedure, as speculated in literature [30]. Coherency of the three population distributions obtained is consistent with the hypothesis that they are biased because of the shared DNA-extraction method [3]. Nevertheless, one cannot exclude that other steps of the sequencing protocol could as well contribute to these phenomena.

### Comparative analysis of recovered *B. fulgida* ORFs

**Figure 3:**
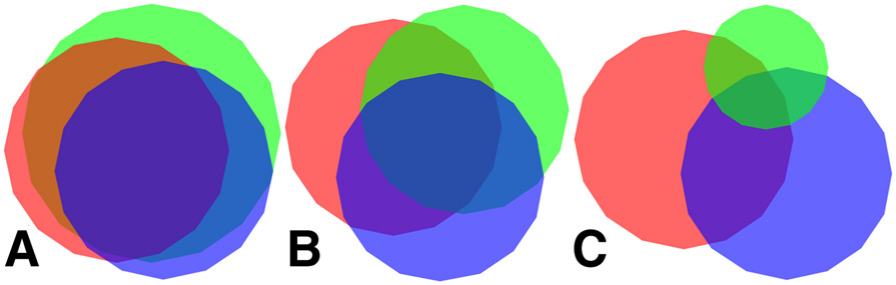
**Generalized Venn diagram of ORFs sets.** Each polygon corresponds to the set of *B. fulgida* ORFs mapped by Shotgun (red), Fosmid (blue), and 454 (green) for a threshold percentage of their length: polygons are displayed for mapping thresholds 0% **(A)**, 50% **(B)**, and 80% **(C)**. In each subfigure, polygons areas are proportional to the number of elements of the sets; proportions between polygons of different subfigures might not respect the actual sizes of sets. This figure was created with VennMaster [31].

According to the BLASTX annotation we performed, 454 recovered many more proteins than the other two technologies (see Additional file 1: Section 2). Specifically, 454 recovered 114.58% and 191.59% more proteins than Shotgun and Fosmid, respectively. However, these differences were smaller when only *B. fulgida* ORFs were taken into account. In that case, 454 recovered 32.71% and 41.49% more *B. fulgida* ORFs than Shotgun and Fosmid, respectively (Additional file 1: Table S3). Similar relations held for the sum of proteins amino acids. The two technologies based on Sanger had similar retrieval performances: they shared about 70% of the recovered ORFs (Figure 3A).

**Figure 4:**
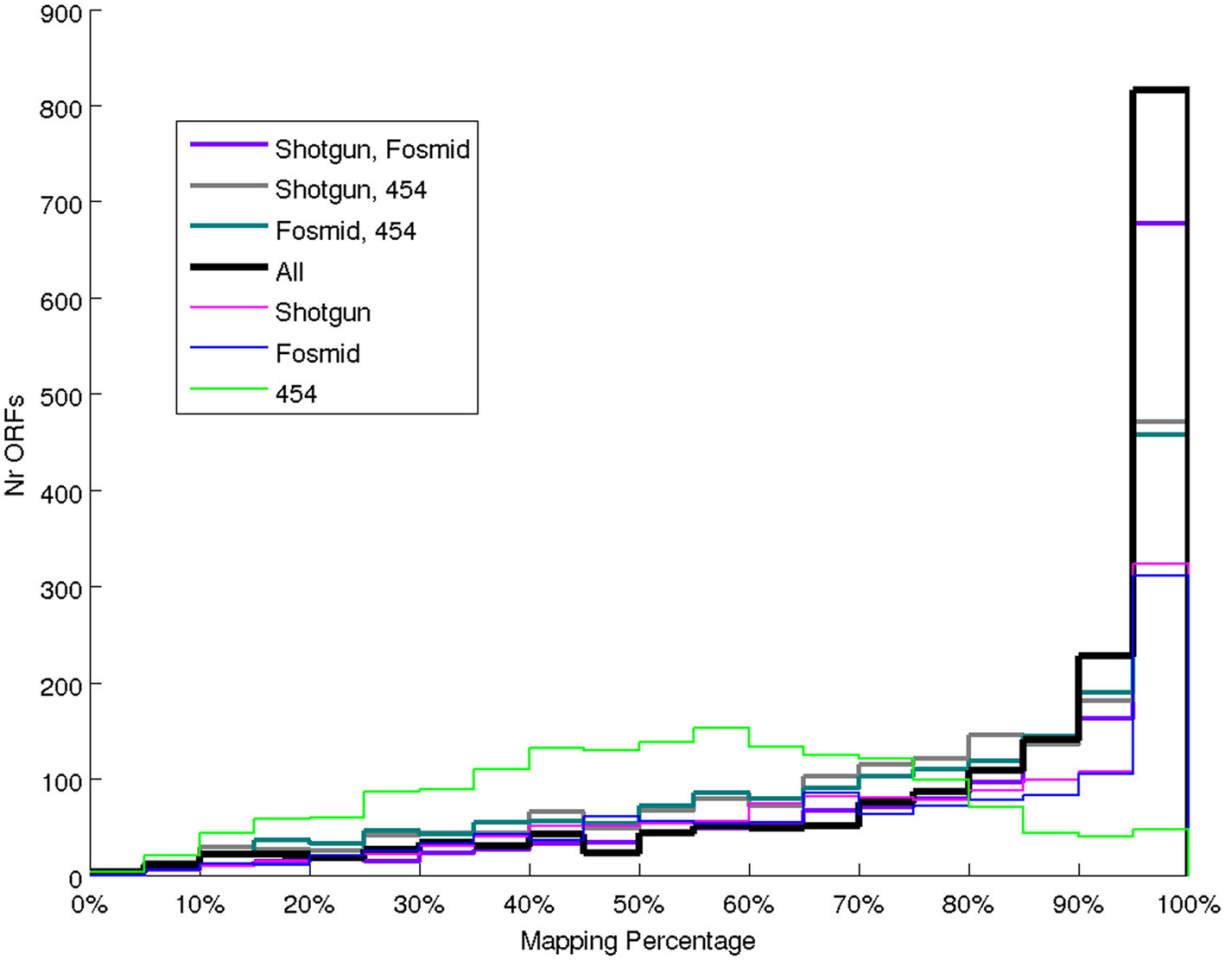
**Number of ORFs with mapping in a given interval.** Histogram of ORF mappings, computed for the *B. fulgida* ORFs recovered by a single technology and combinations of technologies.

Shotgun and Fosmid had similar mapping qualities, as shown by the distributions of recovered ORFs with respect to the size of their recovered parts (Figure 4). In particular, the percentage of the ORFs that they recovered almost completely was remarkably high: for each of the two technologies, about 25% of the recovered ORFs had mapping above 95%. This was probably due to the high average read length (800bp) of Shotgun and Fosmid, that allowed them to recover some ORFs entirely with just one read. Mapping quality of 454 dataset was lower that the ones of the other two: mean and median mapping were both about 54%, and less than 3% had mapping above 95%.

 Comparing the sets of recovered ORFs for different mapping thresholds, we can see that the higher the threshold was, the more the technology biases diverged (see Additional file 1: Section 4). Indeed, the higher the mapping threshold was, the smaller the intersections between sets of ORFs recovered with a feasible mapping by different technologies became (Figure 3, Additional file 1: Table S5). This trend was particularly clear for 454 and it affected its intersections with Fosmid and with Shotgun in the same way. For threshold value equal to 0%, 454 recovered about 90% of each of the sets of ORFs recovered by another technology; for a mapping thresholds of 50% and 80%, this percentage dropped to about 55% and 14%, respectively. The number of recovered ORFs that were shared by Shotgun and Fosmid decreased as well, but at a lower rate. While for a mapping threshold of 0% these two technologies shared about 70% of their recovered ORFs, for mapping thresholds of 50% and 80%, this percentage dropped to about 59% and 38%, respectively.

The coverage variability obtained with different technologies were compared using Pearson correlation coefficient. The correlation analysis of the per-amino acid sequence coverage depths performed on each *B. fulgida* ORF recovered by a pair of technologies indicated that the Sanger-based technologies and 454 coverage patterns were not related (Additional file 1: Figure S2 and Section 3). Indeed, for more than 50% of the ORFs recovered by 454 and Shotgun/Fosmid, the correlation was between -0.3 and 0.3, and hence not significant. On the contrary, there was a significantly positive correlation (above 0.3) for about half of the ORFs recovered by both Shotgun and Fosmid. This indicates that the coverage depths obtained with the two technologies increased or decreased together for the same ORF.

**Figure 5:**
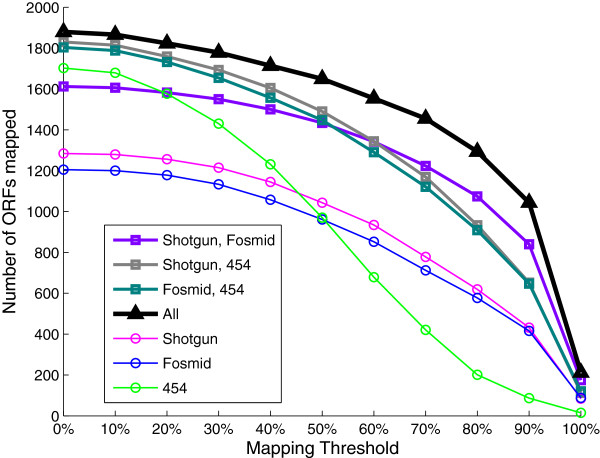
**Number of ORFs with mapping above a threshold.** The plot was computed for the *B. fulgida* ORFs recovered by a single technology and combinations of technologies, for different mapping thresholds.

The fact that different technologies resulted in dissimilar coverage patterns and vastly different sets of ORF with high mapping was observed to be beneficial for improving the ORF recovering. The enhancement was achieved by using together all the reads assigned to *B. fulgida* in the three datasets. The combination of all the three technologies resulted in the recovering of more ORFs than any other combination or any single technology (Figure 5), with a neat increase of the number of ORFs recovered for at least 95% of their length (Figure 4). Using all the datasets together, in particular, the number of ORFs recovered for at least 80% of their length was at least twice the one obtained using the reads of a single technology. A detailed analysis of the effect of combining results from the three datasets is given in the Additional file 1: Section 5.

### Comparative analysis of functional content and ORF location distribution

Functional content distributions based on COG classification did not show significant differences across technologies (Additional file 1: Figure S3). For all the technologies, the most abundant characterized category was COG category C (Energy production and conversion). All the categories related to Information storage and processing (A, J, K, L) were equally abundant. The only category for which there were significant differences was T (Signal transduction mechanisms), that was present in a percentage of less than 2% for 454, and around 6% for the other two technologies.

The location distribution of the recovered ORFs on the putative *B. fulgida* genome was quite uniform (Additional file 1: Figure S4). However, some areas of the genome had a lower coverage depth than the others, and these biases were consistent among different sequencing technologies (Additional file 1: Section 6).

Anyway, these two analyses could be affected more than the others by a potential loss of *B. fulgida* genomic information resulting from the adopted annotation method. Indeed, since *B. fulgida* proteins had not previously been described, we assumed that all reads assigned to the related anammox bacterium *Kuenenia* and all recovered *Kuenenia* ORFs belonged to *B. fulgida*. However, given that the two anammox bacteria are phylogenetically related but not very closely for being two microbes of the same genera [7,32], it might be possible that *B. fulgida* contains ORFs not present in *Kuenenia*. Hence, if these *B. fulgida* ORFs existed, they would not be recovered by our method; in particular, the functional content and the genome location biases would be different from what we found. Nevertheless, as mentioned before, few reads were assigned to other members of B. fulgida’s phylum. Recovering *B. fulgida* information not present in *Kuenenia* through a de novo assembly of the metagenomes can lead to unreliable results, given that the coverage is below 20X [33].

## Conclusions

Anammox bacteria are present in many ecosystems and have important applications in industrial wastewater-treatment. However, genomic information about these bacteria is still very limited. We analyzed the genomic information of the anammox bacterium *B. fulgida* contained in three metagenomes; the metagenomes were acquired from the same community but with different sequencing technologies.

Our analysis indicates that adjustments of sequencing protocols are desirable in order to prevent underrepresentation of *B. fulgida* in the data. This underrepresentation does not seem to be related to a genome location sequencing bias. Sequenced data alone would have given a distorted view of population distributions in the studied community, as observed for other metagenomes [3]. The adoption of PacBio [34] platform could be beneficial for *B. fulgida* genome acquisition, because it seems less biased by GC content.

The population distributions of the three metagenomes were not very dissimilar, despite different sequencing technologies were adopted. This phenomenon is compatible with the hypothesis that DNA-extraction method contributes more to the bias in the population distributions than the sequencing technology [3]. However, one cannot exclude that other steps of the sequencing protocol could as well contribute to the bias; indeed, DNA-fragmentation procedure might have induced the bias toward GC-rich microbes [30]. Nevertheless, our metagenomic data did not allow to directly confirm any of these hypotheses, because the three protocols differ only from the library preparation step onward.

Our results show that the combination of data obtained by different sequencing technologies can allow to recover relevant information of underrepresented organisms. Indeed, even if different technologies recover a microbe in similar abundance, they could do it with significantly different genome-level biases. In our case, technologies coverage patterns revealed to be unrelated for many *B. fulgida* ORFs; moreover, the sets of ORFs recovered by the technologies for a large part of their lengths were vastly different.

## Methods

### Datasets

Metagenome sequencing was performed on three sequencing libraries made from the same DNA sample from the freshwater propionate enrichment described previously [23,27]. Sixty 384-well plates of clones were end sequenced from a 3 kb short-insert Sanger library constructed in pUC18 (henceforth referred as Shotgun), and 62 plates of clones from a 40 kb Fosmid library constructed in pCC1Fos (for detailed library construction and sequencing protocols see [35]). This procedure generated a total of 34 Mb and 30 Mb raw data respectively. A 454 library was also constructed and sequenced on the FLX platform, yielding 59 Mb from 1.25 runs. Raw sequence reads were trimmed with LUCY [36]. The sequences we analyzed are available in DOE JGI Genome sequencing projects database under the name of ’Freshwater-Propionate Anammox bacterial enrichment’, Project ID: 4083784.

Although the size of these data is not very large (Additional file 1: Table S1), it is sufficient for the type of comparative study conducted in this paper. Indeed, data of comparable size were studied in a previous work on the comparative analysis of data generated with different technologies from the same microbial community [3].

With respect to length distribution of reads, a strong similarity between the data acquired by Shotgun and Fosmid could be observed (Additional file 1: Figure S1 and Table S1). The main difference between these two datasets concerned the number of reads they contained: Shotgun acquired about 23% more reads than Fosmid. However, the average length of Shotgun reads was 8% greater than the one of Fosmid. As expected, 454 produced significantly shorter reads than Sanger, but at a higher throughput. The median length of 454 reads was 182bp, about one fourth of the respective value of the other two datasets. The number of reads of 454 was sixfold and fivefold the number of reads of Shotgun and Fosmid, respectively.

### Annotation method

All reads of the considered datasets were submitted as NCBI-BLASTX [37] queries against the NCBI-NR protein sequence database (version of 3 March 2009) [38]. Default BLASTX parameters were used, adding an *E*-value cutoff and a neighborhood word score threshold. Since we wanted to focus only on highly significant alignments, low *E*-value cutoff values were chosen. Specifically, for Sanger-based technologies *E*-value cutoff was set to 10^-6^. As the 454 reads were shorter and the *E*-value of an alignment is directly proportional to the product of the lengths of the two aligned parts, we used for 454 read alignments an *E*-value cutoff of 10^-7^. The word score threshold was set to 14 (default value is 12), in order to increase the speed more than twofold while maintaining a high sensitiveness (see [39], Paragraph 9.3.1.1).

Annotation of reads was based on BLASTX results, adopting what is considered the best stand-alone method [40]: each read was assigned to its best BLASTX hit, at protein and hence at species level. Since *B. fulgida* had not yet been sequenced, its reads could be assigned by BLASTX only to proteins of other organisms present in the reference database. Nevertheless, the reference database we used contained ORFs of another related anammox bacterium, namely *Kuenenia*. Therefore in our analysis we considered all recovered *Kuenenia* ORFs and all reads assigned to these ORFs as belonging to *B. fulgida*.

### ORF recovering: assessment criteria

We used two main quantitative measures to assess the performances of the three technologies with respect to their capability to recover *B. fulgida* ORFs: *per-amino acid sequence coverage depth* and *mapping*.

The *per-amino acid sequence coverage depth* quantifies how well *B. fulgida* ORFs were covered at the amino-acid level by the reads generated by a technology. Specifically, for a technology and an ORF, we considered the reads (generated by that technology) aligned with BLASTX to a particular ORF; the per-amino acid sequence coverage depth of an amino acid of that ORF is defined as the number of times that the given amino acid of the subject ORF was covered by the assigned reads. We considered as covered all the amino acids between the start and the end of a read-ORF alignment. Consequently, if an alignment had gaps, the corresponding amino acids of the ORF were considered covered as well.

The notion of *mapping* measures the part of a *B. fulgida* ORF that can be recovered by the reads generated by a technology. Specifically, the mapping is defined as the percentage of the ORF’s amino acids that were covered (i.e. percentage of amino acids with coverage depth ≥1). Clearly, the mapping can be directly computed from the per-amino acid sequence coverage depths.

For computing the per-amino acid sequence coverage depths and the mapping of ORFs, we considered only those alignments having an identity score greater of equal than 30%. This additional filtering criterion had a very small effect on the recovering performance of each technology (see Additional file 1: Tables S3 and S4).

### ORF Recovering: Comparison Methods

The coverage variability obtained with different technologies were compared using Pearson correlation coefficient. Given two technologies, we considered all the *B. fulgida* ORFs recovered by both; then we computed the correlation of the per-amino acid sequence coverage depths obtained by the two technologies for the same ORF. A similar method for comparing the coverage variability was used in a previous work [5].

We also performed a comparative analysis of the sets of *B. fulgida* ORFs recovered by different technologies. For each technology, we computed the sets of ORFs with mapping above a given threshold; 10 different thresholds were used (0% and all the multiples of 10%).

The sets of *B. fulgida* ORFs recovered by different technologies were also compared with respect to their functional annotation. For each technology, we focused our analysis on the ORFs mapped for at least 70% of their length because we assumed that if an ORF was mapped for such a large part of its length, then all its protein domains could be considered as present in the *B. fulgida* genome. These ORFs were assigned to Clusters or Orthologous Groups of proteins (COG) [41,42] using the Signature web server introduced in [43].

We assessed the improvement achieved by combining different technologies, for pairwise combinations of technologies as well as for the union of all of them. To this end we estimated the resulting *B. fulgida* ORF mapping derived from each technology combination, where an amino acid of the ORF was considered to be covered by a certain combination of technologies if it was covered by at least one of them. Moreover, for each combination of technologies, we computed the sets of *B. fulgida* ORFs with mapping above a given threshold, by varying this threshold as described above.

We performed an analysis to check if sequencing technologies had some location bias in sequencing, i.e., we wanted to examine if some areas of the genome were more covered than others. To this end, we built an approximate representation of *B. fulgida* genome and compared the per-amino acid sequence coverage of the genome obtained with different technologies. The approximate genome was obtained concatenating all *Kuenenia* ORFs in one long amino acid sequence; the ORFs amino acid sequences were concatenated in the same order they are present in the genome of *Kuenenia*. Then, from the ORFs coverage, we computed the per-amino acid coverage of the genome for each sequencing technology.

## Competing interests

The authors declare that they have no competing interests.

## Authors’ contributions

FG participated in conceiving the study and results interpretation, performed the data analyses, and wrote the manuscript. SGT acquired and pre-processed sequence data. GF performed BLASTX annotation. SvH conceived location distribution analysis, designed and participated in carrying out functional content analysis, participated in results interpretation. HOdC conceived location distribution analysis and participated in results interpretation. MJ participated in conceiving the study and in results interpretation. EM participated in conceiving and coordinating the study, analysis of data, and helped writing the manuscript. All authors read and approved the final manuscript.

## Supplementary Material

Additional file 1 **Supplementary Results and Discussion.** Additional figures, tables and description of the obtained results. Click here for file
