# Supplementary material for: Differences in sequencing technologies improve the retrieval of anammox bacterial genome from metagenomes
Source: BMC Genomics. 2013 Jan 16;14:7. doi: 10.1186/1471-2164-14-7 (PMC3618311; doi:10.1186/1471-2164-14-7)
Supplement: Additional file 1 — Supplementary Results and Discussion. Additional figures, tables and description of the obtained results. [file 1471-2164-14-7-S1.pdf]

Supplementary material

1 *B. fulgida* is underrepresented

According to fluorescent *in situ* hybridization (FISH) (Kartal *et al.*, 2008), the *B. fulgida* bacterium constituted about 70-80% of the community. However, less than 14% of the reads generated by each sequencing technology were assigned to *B. fulgida* ORFs, namely 9.68%, 13.76%, and 12.92% for Shotgun, Fosmid, and 454, respectively. Very similar percentages (9.76%, 14.33%, and 11.34%) were obtained comparing the total base pairs of these reads. A rough estimate of the coverage of *B. fulgida* was achieved by dividing the total base pairs assigned to *B. fulgida* by the sum of the lengths of the recovered ORFs, expressed in base pairs (see Tables 2 and 3). Applying this procedure resulted in Shotgun, Fosmid and 454 achieving a coverage of 1.85, 2.14 and 1.29, of the recovered ORFs, respectively.

The fact that about 10% of the base pairs of the sequenced reads were assigned to *B. fulgida* cannot be caused by the presence of relatively large genomes of the other members of the community. Indeed, since at least 70% of the cells of the community belonged to *B. fulgida*, sequenced data would result to be unbiased only if the average size of the genomes of the remaining cells was at least 14 times the size of *B. fulgida* genome. This is impossible because *B. fulgida* genome is likely to be not less than 4Mbp (that is the total size of contigs of its anammox relative *Kuenenia* (Strous *et al.*, 2006)) and the largest known bacterial genome is 13Mbp (Schneiker *et al.*, 2007).

2 454 recovers more proteins and *B. fulgida* ORFs

Table 1 shows that 454 technology sequenced much more base pairs of the others. However, the amounts of base pairs belonging only to the annotated reads were similar: the quantities of annotated base pairs of 454 and Fosmid were almost identical, and a bit lower than Shotgun ones (Table 2). Focusing our attention on *B. fulgida*, we observed that the sets of reads of different datasets annotated to its ORFs had comparable number of total base pairs. Therefore, the performances of these sequencing technologies on *B. fulgida* ORFs recovering could be compared fairly. The percentage of 454 reads that could be annotated was less than what was obtained for the other two technologies; it was 41.71%, whereas for Shotgun and Fosmid was 84.62% and 81.07%, respectively (Tables 1 and 2). This difference was probably due to the short length of 454 reads, increasing the probability of having an alignment by chance and hence not meaningful. Alignments of 454 reads to the reference sequences were more likely to have E-value higher than the chosen threshold, and the higher the E-value is, the more likely it is that the alignment has been obtained by chance.

3 The recovering trend for higher mapping percentage threshold values changes

In this section we compare the sets of *B. fulgida* ORFs recovered by different technologies focusing on the ORFs mapped for at least a given minimum mapping percentage threshold, and on the functional content of highly mapped ORFs.

When the threshold value of minimum mapping was set to zero, the technology 454 gave the best performance, as observed in Section 2. Indeed, 454 recovered more *B. fulgida* ORFs than

the other technologies (Table 3). Moreover, 454 recovered also many of the ORFs recovered by the other technologies: about 90% of the ORFs recovered by Shotgun and by Fosmid (Manuscript Figure 3A), considered separately. Shotgun and Fosmid recovered many common ORFs: the intersection of their sets of recovered ORFs was 877, corresponding to 67.98% of Shotgun and 72.48% of Fosmid. 94.07% of these common ORFs were also recovered by 454.

As the threshold value of minimum mapping was increased, we recognized two trends: the amounts of ORFs recovered by the technologies changed in favour of Fosmid and Sanger, and fewer ORFs were recovered by more than one technology. Indeed, increasing the value of mapping threshold, the number of recovered ORFs decreased faster for 454 than for the other two technologies (Manuscript Figure 5, Table 4). Indeed, as long as the mapping threshold was lower that 50%, 454 recovered more ORFs than the other technologies. From 50% onward, 454 recovered fewer ORFs than the other technologies. This change of relation between the sequences was due to the high number of ORFs that Fosmid and Sanger recovered almost entirely (Figure 4).

In order to better illustrate these behaviours, we compared the sets of ORFs recovered by different technologies for mapping thresholds 50% and 80%. For mapping threshold of 50%, the three sets of recovered ORFs had a symmetric relation. Indeed, they all had about 1,000 ORFs (see Manuscript Figure 5, Table 4) and the intersections between every possible pair of sets contained about 570 ORFs each (Manuscript Figure 3B, Table 5). For mapping threshold of 80%, the set of ORFs recovered by technology 454 was much smaller than those of the other two technologies. Its size was about one third of the sizes of the others (Manuscript Figure 3C, Table 4). 125 of the 201 ORFs of the 454 set were recovered also by at least one of the other two technologies. However, the sets of ORF obtained by Shotgun and Fosmid had similar size and their intersection corresponded to about 38% of each of them. Each of these sets shared about 41% of the ORFs recovered by 454.

4 Shotgun and Fosmid achieve better ORF recovering quality than 454

The similarity between the coverage patterns of a given ORF obtained with two technologies was measured through Pearson correlation. Positive correlation indicates that the coverage depths obtained with the two technologies increase or decrease together; negative correlation indicates that as the depth obtained by one technologies increases, so the depth of the other decreases, and vice versa. The Sanger-based technologies showed similar depth of coverage patterns for 50.29% of the ORFs recovered by both (Figure 2). For 22.17% of the *B. fulgida* ORFs recovered by both technologies, the correlation was between 0.7 and 1; for 28.12% of the ORFs, the correlation was between 0.3 and 0.7. Nevertheless, for 21.59% of the ORFs the correlation was significantly negative (from -1 to -0.3).

In contrast to what happened in the previous case, the Sanger-based technologies and 454 coverage patterns were not related. Indeed, for couples Fosmid/454 and Shotgun/454 the correlations between the coverage patterns did not indicate a significant difference or similarity for the majority of ORFs: for 51.54% and 53.96% of the ORFs, the correlations were between -0.3 and 0.3, respectively. In both cases, the percentage of ORFs with very significant correlation (i.e. outside range -0.7 / +0.7) was less than 10%.

5 Combining all technologies improves ORF recovering

We compared the set of ORFs recovered when considering all the possible technologies combinations. The combination of all the three technologies resulted in the recovering of more ORFs than any other combination or any single technology (Manuscript Figure 5). As a matter of fact, the combination of all the technologies recovered 1879 ORFs, that was 46.26%, 55.93%, and 10.40% more ORFs than Shotgun, Fosmid and 454, respectively; the differences with combinations Shotgun-454, Fosmid-454 and Shotgun-Fosmid were 2.68%, 4.22%, and 16.56%, respectively.

Thanks to the diversity of sequencing biases, combining all the technologies significantly increased the number of ORFs recovered for at least 95% of their length (Manuscript Figure 4). As the mapping threshold value increased, there was an increase in relative gaps between the number of ORFs recovered by the combination of all the technologies and those recovered by any other combination or by any single technology. This trend was particularly strong in comparison with technology 454 and the combinations involving it. These relative differences between the combination of all technologies and Shotgun, Fosmid and 454 alone increased to 108.90% of Shotgun, 124.09% of Fosmid, and 543.28% of 454 at threshold 80%, respectively. The difference of all technologies with combination Shotgun-454 increased to 38.59%; similarly, the difference with combination Fosmid-454 increased to 42.24%. In contrast, the gap between the combination of all the three technologies and the combination Shotgun-Fosmid resulted in a small increase: from 16.56% at threshold 0% to 20.39% at threshold 80%.

Among the combination of two technologies, Shotgun-454 was the best one for low mapping thresholds, while Shotgun-Fosmid was the best for high mapping thresholds. Shotgun-454 combination recovered 1830 ORFs, that is slightly lower than the number of ORFs recovered by the combination of all the technologies. Despite Shotgun-Fosmid was the combination that recovered the lowest number of ORFs (1612), it outperformed Shotgun-454 at mapping threshold of 70% or more.

6 No specific genome location bias of the technologies

We performed an analysis to check if sequencing technologies had some location bias in sequencing, i.e., we wanted to examine if some areas of the genome were more covered than others. Figure 4 shows that the recovered ORFs were almost uniformly distributed on the genome, indicating no strong location bias except for a few spikes. These coverage spikes were mostly consistent among different sequencing technologies, and corresponded to the following ORFs: kustd1658, kustd1783, kustd2042, kuste3701, kuste4036, kuste4355, kuste4640, kuste4642. These ORFs have other almost identical copies in *Kuenenia* genome; moreover kuste3701 differs from kuste4642 for just two amino acids. Therefore, it is likely that BLASTX wrongly assigned to these ORFs many reads that were actually sequenced from their copies. Most of these ORFs corresponded to genes related to DNA replication, recombination, and repair. Some areas of the genome were less covered than the others, and these biases were consistent among different sequencing technologies.

References

Kartal, B., Van Niftrik, L., Rattray, J., Van De Vossenberg, J. L., Schmid, M. C., Sinninghe Damsté, J., *et al.* (2008). Candidatus Brocadia fulgida: an autofluorescent anaerobic ammonium oxidizing bacterium. *FEMS Microbiology Ecology*, **63**, 46–55.

Schneiker, S., Perlova, O., Kaiser, O., Gerth, K., Alici, A., Altmeyer, M. O., *et al.* (2007). Complete genome sequence of the myxobacterium sorangium cellulosum. *Nature Biotechnology*, **25**(11), 1281–1289. 10.1038/nbt1354.

Strous, M., Pelletier, E., Mangenot, S., Rattei, T., Lehner, A., Taylor, M. W., *et al.* (2006). Deciphering the evolution and metabolism of an anammox bacterium from a community genome. *Nature*, **440**(7085), 790–794.

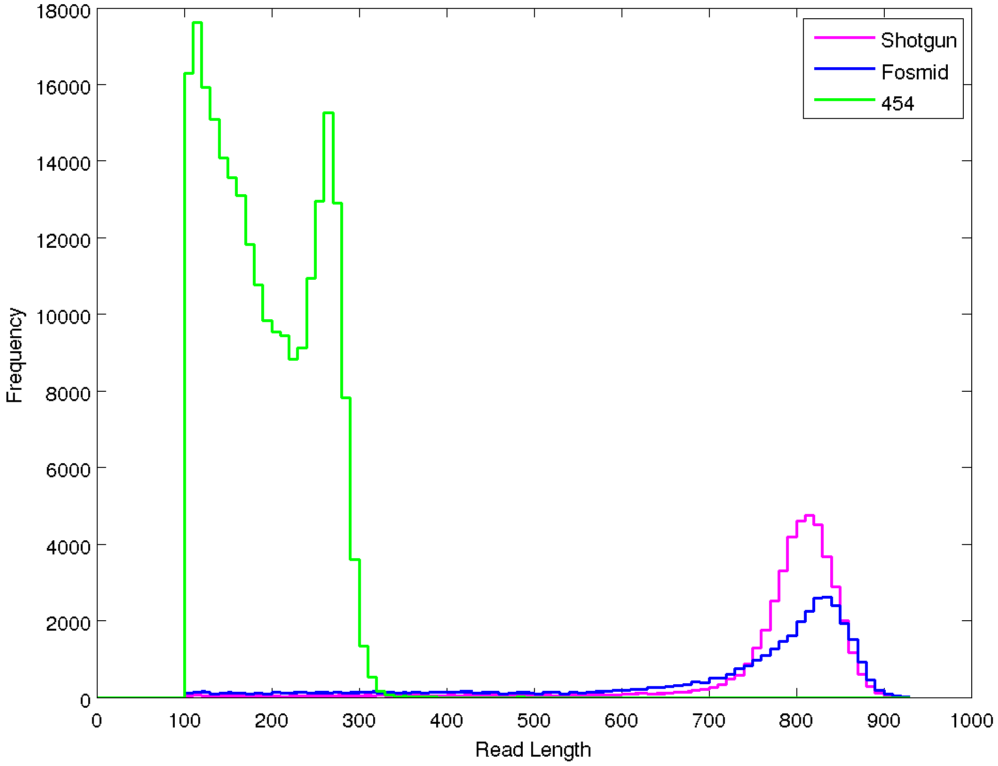

Figure 1: Histograms of length of reads distribution, for each sequencing technology.

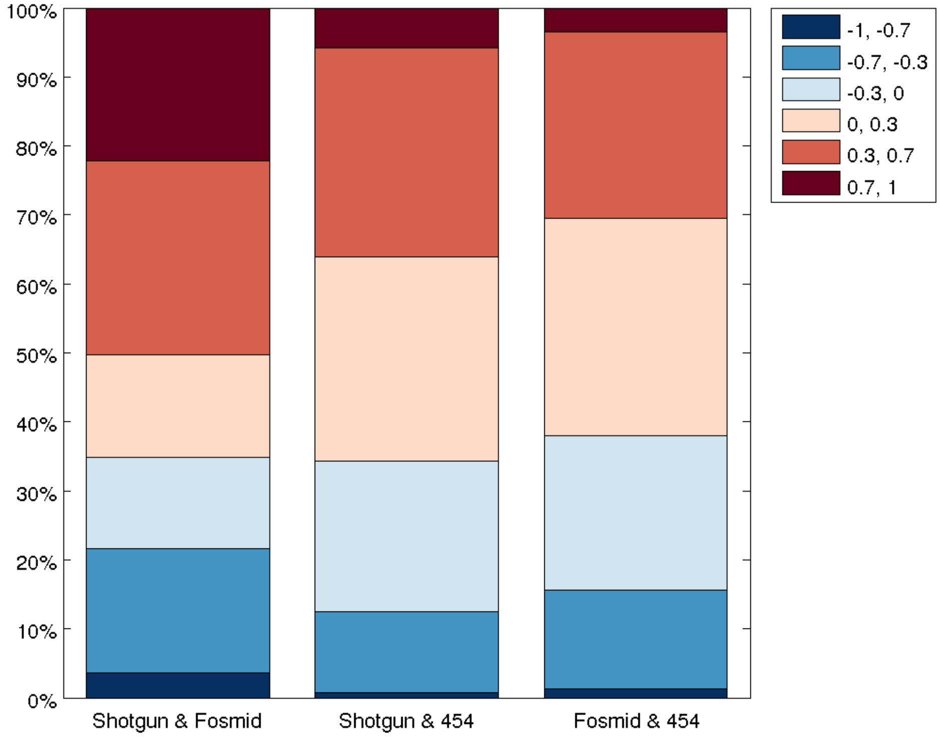

Figure 2: Pearson correlation of depth of coverage patterns. Histograms show percentages of ORFs recovered by both technologies that have correlation within a certain range.

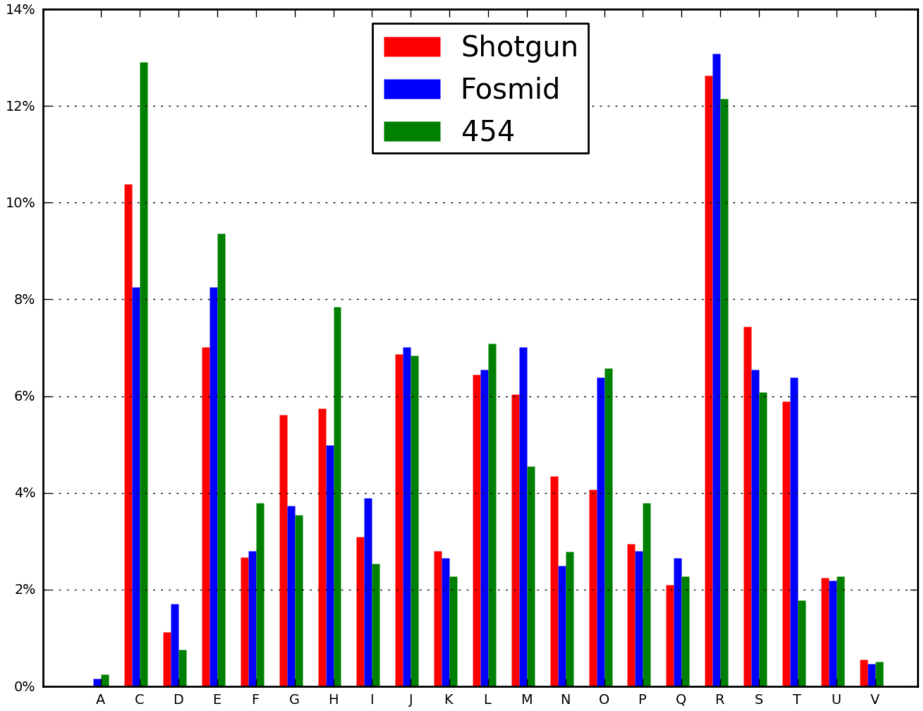

Figure 3: Percentage of COG functional categories of *B. fulgida* ORFs; the analysis is focused on ORFs mapped for at least 70% of their length.

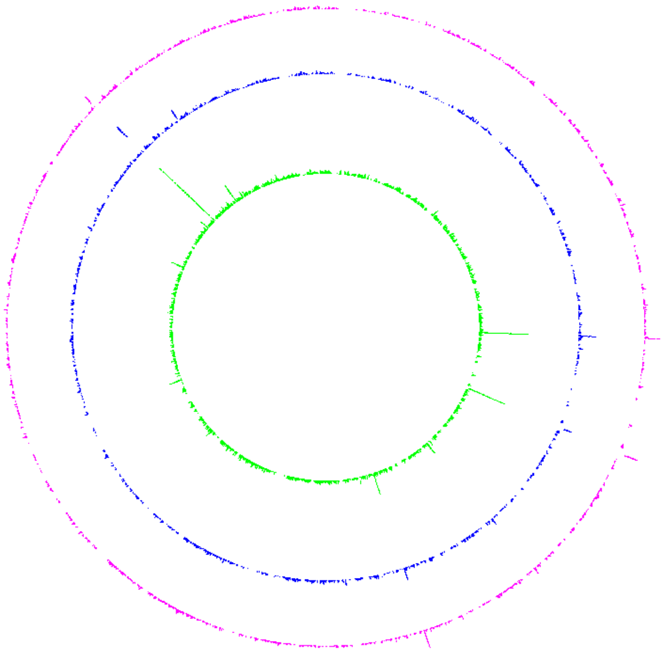

Figure 4: Per-amino acid sequence coverage depth of *B. fulgida* ORFs. The coverage is displayed on the approximate representation of *B. fulgida* genome, obtained concatenating all *Kuenenia* ORFs. The circular plots display the coverage obtained with Shotgun (red), Fosmid (blue), and 454 (green) technologies, respectively.

	Shotgun	Fosmid	454
Number of reads	43,545	35,318	240,646
Total bp of the dataset	34,051,775	25,456,488	45,564,795
Mean length of reads	782	721	189
Median length of reads	806	795	182
Min length of reads	101	101	100
Max length of reads	930	933	482

Table 1: Characteristics of the sequenced data; reads lengths are expressed in base pairs (bp).

	Shotgun	Fosmid	454
Annotated Reads	36,847	28,634	100,382
Reads Assigned to *B. fulgida*	3,568	3,939	12,971
Tot. bp Assigned	29,126,549	21,361,640	21,576,571
Tot bp Assigned to *B. fulgida*	2,841,467	3,061,379	2,445,822

Table 2: Number of annotated Reads.

	Shotgun	Fosmid	454
Proteins recovered	23,868	17,564	51,215
ORFs of *B. fulgida* recovered	1,290	1,210	1,712
Tot AA recovered	11,597,787	8,534,788	24,623,613
Tot AA of *B. fulgida* recovered	512,238	476,444	631,291

Table 3: Number of proteins and ORFs recovered using BLASTX.

Thr.	Shotgun	Fosmid	454	Sho, Fos	Sho, 454	Fos, 454	All
0	1,284	1,205	1,702	1,612	1,830	1,803	1,879
10	1,279	1,200	1,679	1,606	1,814	1,788	1,866
20	1,256	1,178	1,577	1,582	1,759	1,732	1,823
30	1,215	1,133	1,430	1,550	1,693	1,654	1,778
40	1,145	1,058	1,231	1,500	1,605	1,557	1,714
50	1,043	961	970	1,434	1,490	1,447	1,649
60	934	852	679	1,342	1,344	1,290	1,554
70	778	712	421	1,223	1,169	1,121	1,455
80	619	577	201	1,074	933	909	1,293
90	432	416	87	840	652	647	1,044
100	84	88	15	175	112	120	212

Table 4: Number of ORFs recovered by different sequencing technologies and their combinations, for different mapping thresholds. The number of recovered ORFs for threshold 0% is slightly different from the respective number in Table 3 because in this case only alignments with identity $\geq 30\%$ were taken into account. Sho=Shotgun, Fos=Fosmid.

Thr.	Sho∩Fos	Sho∩454	Fos∩454	∩All	Sho∪Fos	Sho∪454	Fos∪454	∪All
0	877	1,156	1,104	825	1,612	1,830	1,803	1,879
10	873	1,145	1,091	816	1,606	1,813	1,788	1,865
20	853	1,078	1,027	766	1,581	1,755	1,728	1,819
30	803	959	914	670	1,545	1,686	1,649	1,772
40	715	787	746	517	1,488	1,589	1,543	1,703
50	593	570	546	344	1,411	1,443	1,385	1,609
60	479	377	355	208	1,307	1,236	1,176	1,462
70	340	201	198	108	1,150	998	935	1,280
80	228	86	81	42	968	734	697	1,044
90	131	37	32	23	717	482	471	758
100	25	8	5	5	147	91	98	154

Table 5: Number of recovered ORFs shared by two technologies A and B (A∩B), number of ORFs recovered by at least one technology among A and B ($A \cup B$), for different sequencing technologies A and B and mapping thresholds. ∩All is the number of recovered ORFs shared by all the technologies; ∪All is the number of ORFs recovered by at least one technology. Sho=Shotgun, Fos=Fosmid.
